# Supplementary material for: Development of Optimized Feed for Lipid Gain in Zophobas morio (Coleoptera: Tenebrionidae) Larvae
Source: Animals (Basel). 2023 Jun 12;13(12):1958. doi: 10.3390/ani13121958 (PMC10295072; doi:10.3390/ani13121958)
Supplement: Supplementary file 1 [file animals-13-01958-s001.zip › animals-2406435-supplementary.pdf]

Supplementary Table S1. The composition of diets containing solidifying materials.

	CP (g/100 g)	CF (g/100 g)	CHO (g/100 g)	Ash (g/100 g)
FW	16.5	8.1	61.4	8.0
FW/A	13.2	6.5	64.1	6.4
FW/C	13.2	6.5	62.1	6.4
FW/S	13.2	6.5	65.7	6.4

FW: food waste; A: agar; C: carrageenan; S: starch; CP: crude protein; CF: crud fat; CHO: carbohydrate
